# The Cost of Universal Suicide Risk Screening for Adolescents in Emergency Departments

**DOI:** 10.3390/ijerph20196843

**Published:** 2023-09-27

**Authors:** Kyle L. Grazier, Jacqueline Grupp-Phelan, David Brent, Adam Horwitz, Taylor C. McGuire, T. Charles Casper, Michael W. Webb, Cheryl A. King

**Affiliations:** 1 Department of Health Management and Policy, School of Public Health, University of Michigan, Ann Arbor, MI 48109, USA; 2 Department of Psychiatry, School of Medicine, University of Michigan, Ann Arbor, MI 48109, USA; ahor@med.umich.edu (A.H.); kingca@med.umich.edu (C.A.K.); 3 Department of Emergency Medicine, School of Medicine, University of California at San Francisco, San Francisco, CA 94143, USA; jacqueline.grupp-phelan@ucsf.edu; 4 Department of Psychiatry, University of Pittsburgh School of Medicine, Pittsburgh, PA 15213, USA; brentda@upmc.edu; 5 Department of Psychiatry, University of Pittsburgh Medical Center Western Psychiatric Hospital, Pittsburgh, PA 15213, USA; 6 Department of Psychology, Harvard University, Cambridge, MA 02138, USA; tmcguire@g.harvard.edu; 7 Department of Pediatrics, School of Medicine, University of Utah, Salt Lake City, UT 84132, USA; charlie.casper@hsc.utah.edu (T.C.C.); michael.webb@hsc.utah.edu (M.W.W.)

**Keywords:** suicide, screening, adolescent, cost analysis, emergency department, universal screening

## Abstract

Suicide is the second leading cause of death among adolescents. As nearly 20% of adolescents visit emergency departments (EDs) each year, EDs have an opportunity to identify previously unrecognized suicide risk. A novel Computerized Adaptive Screen for Suicidal Youth (CASSY) was shown in a multisite study to be predictive for suicide attempts within 3 months. This study uses site-specific data to estimate the cost of CASSY implementation with adolescents in general EDs. When used universally with all adolescents who are present and able to participate in the screening, the average cost was USD 5.77 per adolescent. For adolescents presenting with non-behavioral complaints, the average cost was USD 2.60 per adolescent. Costs were driven primarily by time and personnel required for the further evaluation of suicide risk for those screening positive. Thus, universal screening using the CASSY, at very low costs relative to the cost of an ED visit, can facilitate services needed for at-risk adolescents.

## 1. Introduction

Suicide is the second leading cause of death among adolescents in the US [1]. Emergency departments (EDs) have seen an increasing number of high-risk adolescents, ages 12–17, receiving mental health evaluations, as well as increasing lengths of stay and costs related to these visits [2,3]. Almost 20% of US adolescents visit an emergency department each year [4]. Even before the pandemic, EDs had documented greater numbers of teenagers and young adults experiencing mental health problems and a shift in acuity and diagnoses which have important implications for both ED staffing and outpatient mental illness prevention efforts [5]. The literature during and post-pandemic reinforces the mental health toll on adolescents [6]. For many, the ED is the first contact point to be screened for suicide risk, treated, or referred for needed services [7]. Assessing the cost of screening and the sources of variation in those costs help ED managers, providers, and policy makers determine the feasibility of universal screening for suicide risk.

The novel Computerized Adaptive Screen for Suicidal Youth (CASSY) was used to calculate the costs per adolescent screening. The CASSY was developed and prospectively validated as part of the NIMH-funded Emergency Department Screening for Teens at Risk for Suicide (ED-STARS) study, which was conducted in collaboration with the Pediatric Emergency Care Applied Research Network (PECARN) [8]. The CASSY represents a new development in suicide risk screening as it makes use of algorithms to adapt or personalize screening questions. Youths’ responses to initial screen items determine an initial or provisional estimate of their risk of suicide attempt (application of item response theory); subsequent items are targeted to their risk profile such that the content and number of items vary across youth, resulting in similar measurement precision. The CASSY averages 11 items per adolescent and requires one to two minutes to complete. It provides ED clinicians with youth warning signs (e.g., endorsed suicidal intent) and a continuous suicide risk severity score. This score is an estimate of the probability of a suicide attempt within three months. We used CASSY data from 10 pediatric EDs in the US to model the actual costs of universal suicide risk screening for adolescents who present to the ED with and without a chief behavioral health (BH) complaint.

Universal screening with CASSY provides an opportunity to identify adolescent suicide risk in the ED, providing opportunities for referral and linkage to potentially life-saving services and mental health treatments. The present study is the first to evaluate the costs of implementing the CASSY for adolescents presenting to the ED.

## 2. Materials and Methods

The decision to implement the CASSY, or any screening tool, relies in part on its initial cost to implement the process in the organization or facility, and the ongoing costs of sustaining its use over time [9]. Managerial costing is a conceptual and decision-making framework that characterizes the actual resources and processes in monetary terms. It can also offer insights into the costs of a process [10]. Other researchers have used managerial costing to monetize labor time wages and need estimates of the actual costs within the organization [11]. This approach captures time as a surrogate for effort expended, materials needed to conduct the task, and the wages and benefits for personnel who perform the task. Since the intent was to inform providers and EDs of the cost of expanding suicide risk screening services, primary data on costs for time and materials were collected from the perspective of the hospital emergency department. All research costs were removed from the cost analysis to reflect actual clinical care. Costs incurred by patients, families, or other entities are not included.

An activity micro-costing approach was mapped to a process chart at each site. The process tool identified the site-specific administrative and clinical processes needed to serve an adolescent patient upon arrival to the ED through discharge or hospitalization.

The 10 ED sites differed in the annual average volume of adolescents; geographic location within the U.S.; the number and type of office staff; the use of psychiatrists, ED physicians, nurses, psychiatric social workers, and psychiatric nurses; wages for these personnel by region, city, and state (Core-Based Statistical Area or CBSA); and by time spent by these different ED providers in the process of conducting the evaluation. The costs do not include inpatient treatment and its costs.

The cost study used site-specific data on resources used (time and materials) for ED processes, materials, and personnel to determine the cost per screen per site for adolescents presenting in the ED and capable of being screened. Personnel hourly wages were calculated using CBSA (2020) site-specific and BLS (2020) mean hourly wage by personnel type from the Bureau of Labor Statistics Wage Data by Metropolitan Area (2020) [12]. Wages were adjusted by the 2022 Consumer Price Index (CPI/CPI(U) [13]. Fixed costs related to ED space were not collected from each site. The annual ED cost of CASSY software or integration with an existing EHR are not included in the cost/CASSY. Over 96% of hospitals have an electronic health records system, some with internal personnel who would build the CASSY into a triage ED system; others may have the vendor integrate the CASSY into triage processes or add it to other screens [14].

The cost analysis resulted in the computation of a CASSY screening cost per youth by site. The costs explicitly include a rate of presenting adolescents who are medically capable of being screened; adolescents without a BH chief complaint on arrival at the ED; and the rate of CASSY-positive screens for suicide risk among those adolescents who did not have a BH chief complaint, adjusting for false negatives and positives.

Assessing the cost of screening under universal screening conditions requires measures of sensitivity and specificity of the instrument. King et al. describes the details of the randomized trial and the 2 studies whose data we used in our analysis. This work established that “The CASSY demonstrated a high AUC for the prediction of an SA, with an excellent balance of sensitivity and specificity, and is suitable for administration in busy EDs”.We applied the estimates from Study 1 to our stepwise count of adolescents from Study 2 to calculate costs. In other words, costs were a function of the presenting condition, the result of the CASSY (CASSY produces a dimensional risk estimate) and other variables, and the number of adolescents treated at that stage of the process flow. This means that costs were estimated by taking sensitivity and specificity into account.

Because the cost study was based on the Study 2 sample that was enhanced for research purposes, the cost/youth is weighted by the data from ED-STARS Study 1, in which all presenting youth who were capable of being screened were screened with the CASSY. Study 1 provided rates of youth presenting with and without a BH chief complaint and the rates per site of youth with high-risk scores from the CASSY among those with and without a BH condition upon arrival.

**Figure 1:**
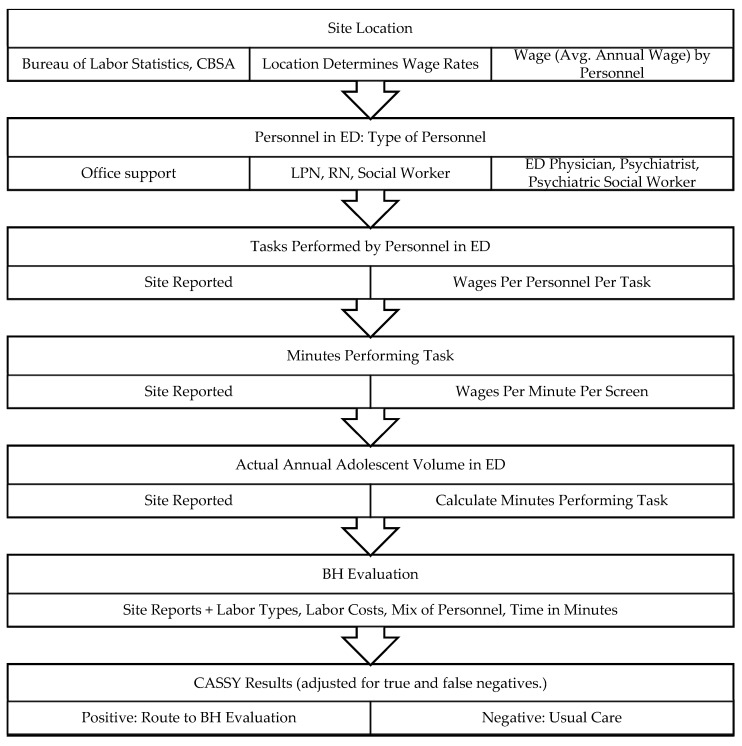
Flowchart of calculation of personnel time cost per CASSY per site. Note: tablet-based screen; office support staff administer screen; staff reports screen results to RN. Definitions: ED: emergency department. BH: behavioral health. LPN: licensed practical nurse. RN: registered nurse. CBSA: core-based statistical area, a Bureau of Labor Statistics unit of population.

This study uses an ED patient flow model to identify costs associated with screening from the point of entry to the ED through discharge [15] (See Figure 1). Data on presenting condition, facility size (volume of adolescents), personnel types and tasks, and time costs are derived from ED-STARS Study 1 (universal screening) and Study 2 (actual implementation costs of time, personnel, materials), conducted in collaboration with PECARN.

Costs were weighted by the rate of ED use by presenting adolescents with and without BH conditions, medical capacity to be screened, and the rate of CASSY identification of high suicide risk among those without a BH condition on arrival. The cost model used ED-STARS Study 1 data to identify numbers of adolescents presenting at the ED, and the likelihood of screening positive on the CASSY, with and without BH conditions]. Site-specific data from Study 2 were collected on labor rates for ED personnel, ED staffing, task times in minutes, and annual adolescent volume. Site-specific non-research-related costs and national statistics data provide costs of implementing and administering the CASSY. The model incorporates the higher costs incurred when suicide risk is detected among adolescents. Follow-up for CASSY-positive screens usually includes a psychiatric or mental health evaluation conducted by specific personnel in the ED; we captured the actual time and wages for the personnel. These costs are evaluated using site-specific and national data to determine the financial feasibility of implementing CASSY as a universal screening tool for adolescents presenting in EDs.

## 3. Results

Adolescents with and without a BH chief complaint, and those with a chief complaint of suicide ideation or a suicide attempt, are assigned a risk category when screened by CASSY [16]. We found some variation across ED sites in the percentages of adolescents who (a) presented with a BH chief complaint and screened positive for high suicide risk (8.43% to 31.97%); (b) presented without a BH chief complaint yet screened positive for suicide risk (0.95% to 5.03%).

The average cost per adolescent was USD 5.77 for universal screening and USD 2.60 for the screening of adolescents who presented without a behavioral health (BH) complaint. The average total cost per CASSY ranged by site from USD 3.87 to 7.53 (Avg. USD 5.77). The higher cost of USD 7.53 is associated with the conservative assumption that no suicide risk screening would have been conducted if the CASSY were not administered, even for adolescents presenting with a BH complaint (including suicide risk). In a model that includes only the costs for those who present without a BH complaint, the average cost per CASSY ranged from USD 1.96 to 3.20 (Avg. USD 2.60) per adolescent screened. This represents the incremental cost of administering the CASSY and detecting suicidal risk among adolescents who otherwise may not have received a mental health evaluation in the ED.

Table 1 shows selected characteristics of the sites, proportion of adolescents who present without a BH chief complaint but who are identified as at risk on the CASSY, costs per adolescent screened, and costs encompassing screening implementation, including administration, review, and follow-up with positive screens. This average cost per site accounts for the cost of treating adolescents who present without an initial psychiatric chief complaint but who screen as high risk on the CASSY, with a subsequent evaluation. This cost includes all stages of the clinical process in the ED and their associated costs. The mean cost per screen is USD 2.68.

For example, for Site 1, the average incremental cost of CASSY screening among those for whom screening is medically possible when presenting without a BH complaint but screening as high risk is USD 2.41 per screen.

Cost per Adolescent Screen—BH and Non-BH show the total average cost of screening using CASSY under conditions of universal screening. This cost includes the cost of screening those with and without a behavioral health chief complaint on presentation, and follow-up MH evaluation costs. The mean cost is USD 5.77 per screen.

Costs do not include the initial infrastructure cost of either integrating the screening tool into the electronic health record or paying an annual software user fee. Estimates for these two setup options are described in the Discussion.

The final model estimated CASSY costs for universal screening—screening of all presenting adolescents. The percentage of adolescents who present to the ED with a non-BH chief complaint, but who then screen as “high risk” on the CASSY, slightly increased the average cost per CASSY. In the ED with the highest percentage of adolescents in this category (5%), the average total cost was USD 3.20 for non-BH screens. If this ED were considered an outlier, and not included in the model, the correlation is significantly reduced. The initial CASSY administration cost is the same for adolescents who present with and without BH chief complaints; however, because a higher percentage of adolescents with BH chief complaints screen positive on the CASSY, necessitating mental health follow-up evaluations, BH chief complaints increase the overall average costs. Table 2 shows the percent of adolescents who screened as high-risk among those who presented with and without BH complaints.

## 4. Discussion

EDs are a common point of access for mental health services among adolescents, and ED visits for adolescent suicide risk have more than doubled in recent years [17]. Medical EDs can adopt universal adolescent suicide risk screening as part of existing screening, intake, and triage processes. Moreover, the processes are already in place for screening at accredited EDs, as the Joint Commission requires suicide risk screening for patients, ages 12 and older, who are evaluated or treated for behavioral health conditions [18]. They also recommend suicide risk screening for all patients [19]. Given the worsening mental health of our nation’s adolescents, exacerbated by the COVID-19 pandemic and a declaration of a national emergency [20], such screening is especially timely.

According to the Medical Expenditure Panel Survey (MEPS), the average cost of an ED visit was USD 1150 in 2020, up 6.3% from 2019; universal CASSY suicide screening of adolescents would add little to ED budgets under most staffing configurations [21]. The screening takes 1–2 min, with additional staff time and costs associated with conducting mental health follow-up evaluations for those youth who screen positive. Average costs of the CASSY may also decrease as operational processes improve. Regarding the initial and ongoing infrastructure costs associated with the CASSY, institutions have the option of integrating the CASSY into their electronic health record (EHR) or paying an annual user fee. If integrated into the EHR, the cost would be composed of the institution’s cost to integrate a screening tool into their EHR, an annual IT maintenance fee, plus an estimated USD 0.30 to USD 1.00 per screening, depending on ED volume. If the institution chooses to link with an external company’s portal, the estimated annual IT cost for setup and use may be approximately USD 10,000.

These cost analyses, based on use of the CASSY, are reasonable estimates for universal screening in the ED using other screening tools, with some caveats. The cost of administration (handing out the screening tool and checking screen results) would be expected to vary minimally across screening tools. However, because most costs relate to personnel costs for mental health evaluations with adolescents who screen positive, the costs associated with other tools could differ based on the tool’s sensitivity and specificity, the positive screen threshold, and whether one- or two-stage screening protocol is used. They may also vary based on who conducts the follow-up evaluations with adolescents who screen positive and with the length of these evaluations.

We recommend a standardized universal suicide risk screening protocol in EDs because such a strategy identifies adolescents at risk for suicide who do not present with suicide risk and may benefit from mental health services [22].

The CASSY has notable strengths that include its excellent overall prediction accuracy; it provides ED providers with useful information, including probability of risk for a suicide attempt and a list of suicide risk warning signs endorsed by the patient; and it offers ED systems to customize a positive screen threshold (balancing specificity and sensitivity). The time needed for an adolescent to complete any of a variety of suicide risk screens is minimal; the costs are driven by the necessary involvement of ED personnel.

Although these results show the low cost of screening youth for suicide risk using CASSY, there are limitations. These results may not be generalizable to all US EDs. The characteristics of the study EDs differ from many general and specialty EDs. The availability of the different types of ED providers, processes for intake and evaluation of presenting youth, and the supply of other nearby sites for emergency care differ by locale, ownership, and availability. Organizational efficiencies in delivering screening may increase over time, reducing unit and total costs. As noted earlier, information and medical record technology affect ease of implementation and total costs. Managerial cost data were collected cross-sectionally (adjusted to 2022 values), at one point in time at the sites, rather than longitudinally.

This study did not assess the outcomes of screening and follow-up, cost effectiveness of CASSY screening, or downstream impacts of lack of screening. While downstream healthcare costs of screening are generally greater than the cost of screening, not all EDs have the personnel and resources to respond to youth who present with these challenges.

## 5. Conclusions

The CASSY is a validated screening instrument for adolescent suicide risk, and the actual monetary costs of using the CASSY to screen all adolescents presenting in the ED are low, with most costs being associated with mental health evaluations for adolescents who screen positive. Universal screening facilitates the recognition of adolescent suicide risk in the ED, providing opportunities for referral and linkage to potentially life-saving services and mental health treatments. 

## Figures and Tables

**Table 1:** Screening costs and selected characteristics of EDs by site.

Site	Cost per Adolescent Screen: BH and Non-BH	Cost per Adolescent Screen: Non-BH Only	Avg Annual ED Volume (12–17 yrs.)	ED Site Location (USA)
1	USD 6.57	USD 2.41	<10,000	Northeast
2	USD 4.69	USD 2.83	>16,000	Mid-Atlantic
3	USD 5.40	USD 2.43	>16,000	Midwest
4	USD 7.53	USD 2.44	10,000–16,000	Mid-Atlantic
5	USD 6.16	USD 2.62	10,000–16,000	Mountain West
6	USD 5.08	USD 2.22	10,000–16,000	Northeast
7	USD 6.43	USD 2.28	10,000–16,000	Midwest
8	USD 3.87	USD 1.96	<10,000	Midwest
9	USD 5.33	USD 2.63	>16,000	Midwest
10	USD 6.66	USD 3.50	<10,000	Mountain West

Note: BH: Behavioral Health. Three study sites were omitted from cost analysis due to low number of study participants. Actual annual volumes per site (2017–2018) were used in the model; to mask site identification, categorical variables appear in the table. Costs adjusted for sensitivity and specificity from ED-STARS.

**Table 2:** Percent screening as high-risk and presenting with and without behavioral health chief complaints among all adolescents by site.

ED Site	% BH Complaints and Screen High Risk	% Non-BH Complaints and Screen High Risk
1	1.86	0.00
2	2.40	4.32
3	8.44	3.62
4	10.95	1.26
5	11.33	2.61
6	5.70	2.62
7	2.83	2.02
8	13.06	3.13
9	6.01	1.39
10	12.34	5.65

## Data Availability

Data are being prepared for open availability in a publicly accessible repository. In the meantime, data are available from the senior author (C.A.K.).
